# Basic and target eco-environment water requirements of a dry inland river under typical flow frequencies in China

**DOI:** 10.7717/peerj.8285

**Published:** 2020-01-02

**Authors:** Aihong Fu, Weihong Li, Yi Wang, Yifei Bai

**Affiliations:** 1 State Key Laboratory of Desert and Oasis Ecology, Xinjiang Institute of Ecology and Geography, Chinese Academy of Sciences, Urumqi, Xinjiang, China; 2 University of Chinese Academy of Sciences, Beijing, China

**Keywords:** The runoff, Ecological base flow, Based eco-environment water requirement, Target eco-environment water requirement, Desert riparian forest, Typical water frequencies, The guarantee degree, Water resource allocation, The Yarkand River, The arid region

## Abstract

Analysis of eco-environmental water requirements (EEWRs) and water resource allocation strategies for arid, inland river basins can provide the theoretical basis for sustainable water utilization and management. In this paper, an optimal water resource allocation strategy is proposed for Yarkand River Basin in Xinjiang, China, on the basis of a comprehensive analysis of runoff data collected between 1970 and 2016, three ecological environmental protection goals, basic eco-environmental water requirement (BEEWR) aimed at sustaining aquatic ecosystems within the river, and target eco-environmental water requirements (TEEWR) aimed at protecting various types of riparian vegetation along the river. The results showed that: (1) after the runoff in Kaqun reach subtracting the BEEWR, the annual average river loss (recharge), and the amount of water diversion for irrigation (51.43 × 10^8^ m^3^) from flows along the Kaqun reach, the remaining water volume during wet years was able to meet all three TEEWRs; (2) during moderately wet years, the remaining water was capable of meeting the second and third TEEWRs; and (3) during dry and extremely dry years, there was little or no residual water available to meet TEEWRs. The proposed optimal water resource allocation strategy, based on the above findings, states that the water diversion requirement for irrigation and domestic use allocated from the total amount of runoff should not exceed the National Water Policy (Three Red Lines) standard first. Then, the BEEWR allocated from the runoff should be met second, and the annual average river loss, third. Depending on the amount of remaining water, the second and third TEEWRs can be fulfilled during wet years, but during moderately wet years, only the third TEEWR can be met. During dry and extremely dry years, only the BEEWR of the river can be met and only during the flood season.

## Introduction

The eco-environmental water requirement (EEWR) is the total amount of water resources required to maintain a particular ecosystem. EEWRs have been based on multiple parameters including the water needed for the protection and restoration of natural vegetation and aquatic ecosystems associated with inland rivers (Wang et al., 2001; Ni et al., 2002). Due to its specificity, EEWRs are suitable for systems analyses of arid, semi-arid, and seasonally arid, semi-humid areas, in which the contrast between water supply and demand is prominent, and the ecological environment is fragile. During this study, the EEWRs of the Yarkand River Basin in Xinjiang, China were examined, including the water demand needed for ecosystems both within and outside of the river.

The water demand for the river is divided into two kinds of EEWRs: basic and target water requirements. A basic EEWR (BEEWR) refers to the minimum amount of water that needs to be retained in the river to maintain the eco-environmental function of the river’s aquatic ecosystem (Ling et al., 2014). A target EEWR (TEEWR) refers to flows required to meet the protection goals of ecosystems outside of the river, such as riparian vegetation.

Previous international studies have focused on the minimum EEWR (Franchini, Ventaglio & Bonoli, 2011; Shokoohi & Hong, 2011; Tran et al., 2011), the minimum ecological flow (Yang et al., 2012; Zhang et al., 2019), and the reasonable ecological flow (Chen et al., 2012) that is needed to maintain the survival of aquatic organisms and/or improves the aquatic environment. However, none of previous studies considered simultaneously the use of water for agricultural irrigation or industrial and domestic use, and the protection of riparian forests and vegetation. However, some researchers have studied the balance between agricultural and ecological water use in a basin, and found that achieving such a balance is an enormous challenge (Koutrakis et al., 2018; Cheng & Li, 2018). In terms of measures for ecological flow protection, these studies contended that ecological flows can be supplemented by the control and dynamic distribution of various water sources (Yan et al., 2018). In a hyper-arid inland river basin, river waters are not only used to ensure the health and stability of riverine ecosystems, but are also used for agriculture and to supply the ecological water demand of riparian vegetation. Therefore, previous quantitative studies of EEWRs for rivers should be augmented in order to: (1) meet both agricultural irrigation and ecological protection requirements of desert riparian forests (Han et al., 2010); (2) provide guidance and applied research on the water needed to maintain ecosystem functions (Shokoohi & Amini, 2014); and (3) strengthen ecological water requirements for water resource allocation (Teaf & Kuperberg, 2004). The findings of these investigations can provide a scientific basis for the rational allocation of water resources, while protecting and restoring the ecological environment.

With the increasing shortage of water resources, the reasonable allocation of water based on quantified EEWRs has become a major field in the study of ecological, economic, and social sustainable development of watersheds (Zhang, 2017). At the domestic level, only a few researchers have considered riverine flows that meet the needs of industrial and agricultural production as well as domestic water supplies in a basin (Zhang, 2011; Gao, 2018; Liang, 2018), and/or that meet the purposes of social and ecological protection of rivers (Wang & Lu, 2009; Tang, 1995; Ferreira & Teegavarapu, 2012; Khamidov, 2007; Khudaiberganov, 2007). Moreover, if an especially dry year is encountered, water resources are extremely scarce, and it is difficult to simultaneously achieve economic, social, and ecological benefits from the available water. To this end, it is necessary to identify the requirements for socio-economic and ecological water use given the differences that typically occur in total annual runoff for a given recurrence/flow frequencies, and put forward ecological protection goals for different types of runoff years.

The Yarkand River is a typical inland river in China. The river originates in the Karakorun Mountains and flows are primarily derived from snow (ice)-melt. The seasonal differences in flow in the river are significant. Flow during the summer is large, whereas winter flows are limited. Importantly, the Yarkand River basin is host to more than 1.96 million people in southern Xinjiang. Moreover, water in the river is used to irrigate approximately 0.60 million ha of agricultural land. In fact, agricultural water comprises 98% of all water use. Irrigation is primarily conducted using traditional flood irrigation methods; more efficient, low-water use irrigation is limited. Since the efficiency of water use is extremely low, agricultural water use seriously affects the amount of water that is available to support ecological needs. The Yarkand River is also one of the main tributaries to the Tarim River. In recent decades, the increased population in the region, combined with the increasing use of water for irrigation has resulted in a complete drying of the river before reaching the Tarim River. Indeed, the Yarkand has not discharged water into the Tarim River since 1986. Consequently, it lost its function as a surface water source for the Tarim River (Xu et al., 2014).

Large areas of desert riparian forest vegetation also exist along both sides of the Yarkand River. Due to the large diversions of water to irrigated areas, overbank riverine flows on which natural riparian vegetation depends have been cut off, resulting in the large-scale degradation of desert riparian forests, soil erosion, and desertification (Chen et al., 2014). Indeed, the function of the riparian ecosystem has been seriously reduced. For this reason, the Yarkand River not only needs to meet the flow requirements for irrigation within the basin, but must also maintain EEWRs in the Tarim River and the requirements of natural desert forest vegetation along both rivers (Bai, Xu & Ling, 2014). Overall, it is necessary to control water diversions to irrigated areas, maintain ecological base flows in the river, and rationally allocate the remaining water resources to ecologically protect the river basin as much as possible (Zhou et al., 2012).

To effectively control the diversion of water from irrigation areas in river basins, the Chinese government has issued a “stringent national water resources management system and the Three Red Lines policy” (He, 2017). This National Water Policy requires all regions to document the total amount of surface water use in river basins as soon as possible. In other words, water diversions to irrigated areas should not exceed the total amount of surface water use (red line). The total amount of surface water used to maintain the irrigation and domestic use of the Yarkand River basin (its red line) is 51.43 × 10^8^ m^3^. Given that the total amount of surface water in the basin is maintained below the Three Red Lines during typical years of both abundance and drought, it is not known whether the remaining water, after the runoff deducting for river loss (including recharge and evaporation), the total amount of surface water use (red line) and the BEEWR, will be able to fulfill the TEEWR of the river or not.

The aim of this article is to propose a water resource allocation strategy based on a comprehensive assessment of the water needed to meet industrial and agricultural production, domestic water needs, and ecological water requirements within the Yarkand River basin. The analysis is based on runoff data collected between 1970 and 2016 at a monitoring station along the Kaqun reach of the Yarkand River, three previously defined ecological environmental protection goals, and both BEEWR and TEEWRs for different recurrence frequencies of total annual runoff. Subsequently, a water resource allocation strategy is proposed on the basis of the residual (remaining) water volume after the runoff deducting the total amount of surface water use (red line), river losses, the BEEWR, plus other withdrawals, to ensure the sustainable utilization and management of water resources in the basin. The strategy also provides a reference for inland river water resource management in other arid zones of the world.

## Study Area and Methods

### Study area

**Figure 1:**
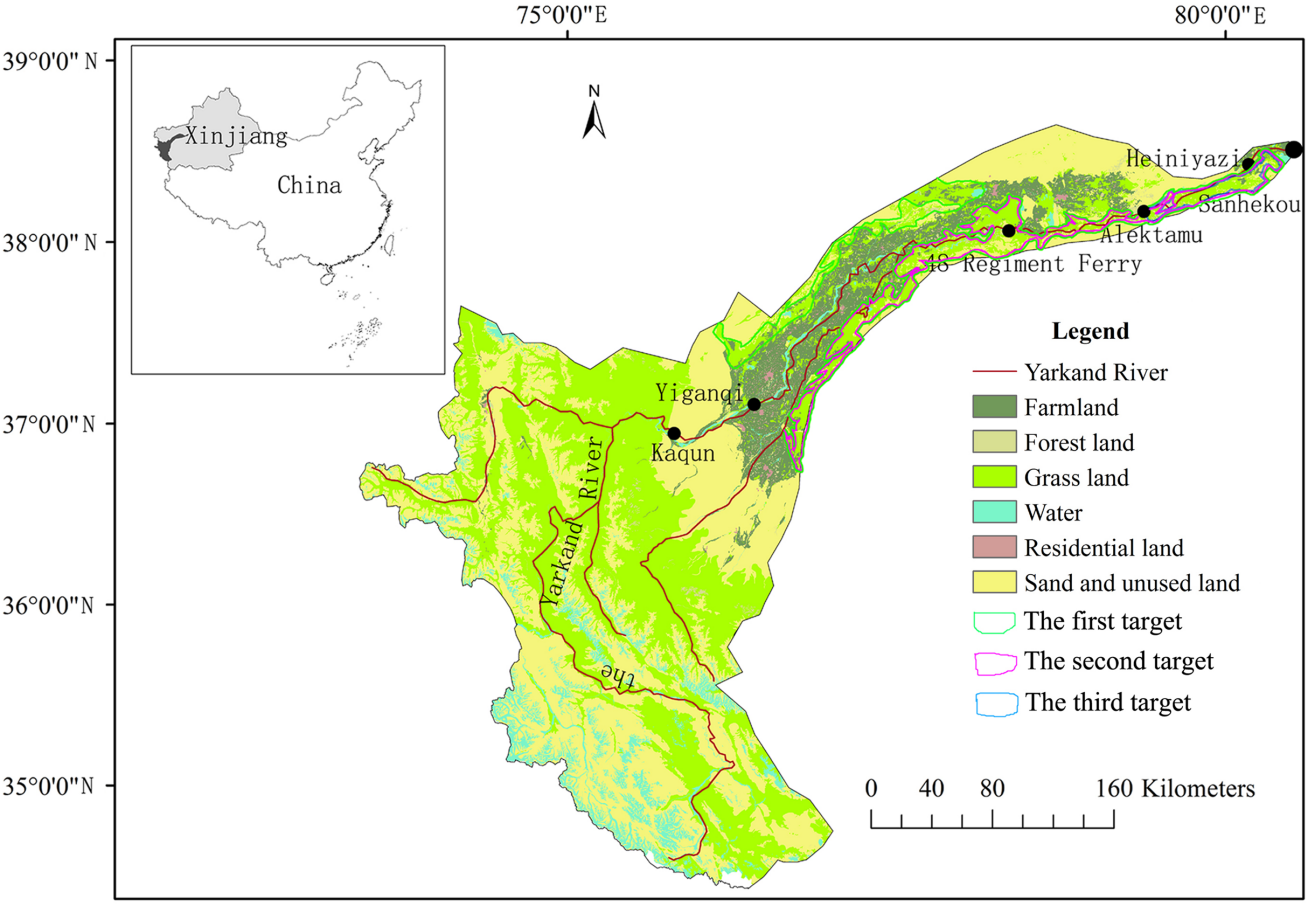
Map showing the location and the primary land-use/land-cover categories of the Yarkand River Basin in China.

The Yarkand Basin (Fig. 1) is located in the southwestern portion of the Xinjiang Uygur Autonomous Region and the southwestern section of the Tarim Basin. The basin is bordered by the Taklamakan Desert to the East, the Tuogelake and the Gaya Deserts to the West, the Karakoram Mountains to the South, and to the south of the Tianshan Mountains. The total basin area is 8.57 × 10^4^ km^2^ (excluding 8.44 × 10^4^ km^2^ in Kashmir and Afghanistan due to the inability to use this part of the water). Topographically, the mountainous area encompasses 5.84 × 10^4^ km^2^, and accounts for 66.9% of the basin. The alluvial plain covers 2.73 × 10^4^ km^2^, accounting for 33.1% of the basin.

The Yarkand River is one of the largest inland rivers in China and the main source of water to the Tarim River. It originates from the north side of the southern Karakoram Mountains. The Yarkand River water system includes the Yarkand River, the Tiznafu River, the Kekeyahe River, and the Uruk River. The Yarkand River has a total length of 1,281 km, with an average annual runoff of 72.04 × 10^8^ m^3^ (in 2000–2016). The Tiznafu River was a tributary of the Yarkand River, and its average annual runoff is 8.3 × 10^8^ m^3^. The average annual runoff for the Kekeyahe and Uruk Rivers is 0.89 × 10^8^ m^3^ and 0.08 × 10^8^ m^3^, respectively. The Yarkand River consists of five hydrographic sections, including the Kaqun, Yiganqi, the 48th Regiment Ferry, the Alektamu, and the Heiniyazi. The Kaqun is located near the mouth of the Yarkand River and contains the downstream most monitoring station.

The Yarkand River Basin is characterized by an arid continental climate with little rain and strong evaporation. The average annual temperature is 11.7 °C, and annual rainfall is 61.5 mm.

The Yarkand River Basin economic zone is mainly concentrated in the plains irrigation area, which is an area where water is controlled by Yarkand water system. Its upper reaches are located in Pishan County in the Hetian area and the lower reaches within the three banks of Awati County in the Aksu region. In 2010, the total irrigation area encompassed 58.70 × 10^4^ ha. As of 2011, the total population of the Yarkand River Irrigation District was 196.01 × 10^4^ people. There are 76 trunk canals and sub-trunk canals in the irrigation area; their total length is 1,704.6 km. In 2017, the total annual drainage volume of the river was 59.00 × 10^8^ m^3^, accounting for 86.56% of the average annual runoff at the Kaqun station. Limited water resources in the Yarkand River Basin, along with the disordered clearing of land, over-designated water use, and the seizure of ecological water are prominent, resulting in increased conflicts concerning water use. As a result, downstream water rights are difficult to maintain and guarantee. According to previous investigations (Fu, 2008; Yu, 2002), there was essentially no water flowing from the Yarkand River into the Tarim River in 1986. In addition, the upstream allocation of water resources was met first, before water moved downstream. In years characterized by abundant water, the water was transferred downstream under the premise that the remaining water would be used for socio-economic development in downstream regions. Only after a dry water source area meets the basic water demand can water be transferred downstream. Overall, the limited amount of available water cannot meet the water demands of the downstream areas.

### Methods

#### Data collection

##### Runoff along the Kaqun reach

Monthly runoff along the Kaqun reach (as shown in Fig. 1) was monitored in 1970–2017 by the Kashi Authority of the Tarim River Basin, China.

##### The annual average BEEWR

The BEEWR at the Kaqun monitoring station and at other reaches was calculated by the improved Tennant method (Mishra, Amrit & Pandey, 2019). The BEEWR of the Yarkand River was 12.08 × 10^8^ m^3^. The BEEWR of the Yiganqi reach (for the entire year), the 48th Regiment Ferry (April to September), the Alektamu reach (June and September), and the Heiniyazi reach (September) were 9.31 × 10^8^ m^3^, 2.71 × 10^8^ m^3^, 1.19 × 10^8^ m^3^, and 0.44 × 10^8^ m^3^, respectively. Because BEEWR is the minimum flow maintaining the health of the river, the Yarkand River is a seasonal river, in the non-flood season, it is still necessary to retain a large amount of water to meet the local people’s production and domestic water use. The remaining water is difficult to meet the minimum flow of the river throughout the year, especially the lower reaches of the river. Therefore, for the Yarkand River, below the 48th Regiment Ferry, the remaining water only needs to meet the BEEWR during the flood season or a certain month. BEEWR did not change with total annual flow frequencies in the previously described reaches.

##### Environmental protection goals and ecological water requirements

Given the season and vegetation characteristics of seasonal rivers, as well as the requirements for ecological water transmission to the Tarim River, three hierarchical ecological protection goals (Fig. 1) were proposed for the area (derived from the Xinjiang Tarim River Basin Kashi Management Bureau). The first protection goal was based on the “ecological protection red line” policy implemented by the Chinese government (Huang & Yan, 2017). The ecological protection red line for natural vegetation in the Yarkand River Basin had been designated as 74.29 × 10^4^ ha. The second protection target was related to the area covered by ecologically sensitive natural vegetation along the lower reaches of the Yarkand River, which totaled 22.27 × 10^4^ ha. The third protection target focused on key types of vegetation, including *Populus euphratica* forest areas with ecologically sensitive areas. These forests were mainly distributed along both sides of the 320-km-long river channel from Alektamu to Sanhekou (Fig. 1). *P. euphratica* forest covered 0.09 million ha.

Typically, the ecological water demand for vegetation is calculated using the Area Quota Method or the Diving Evaporation Method (Ye, Chen & Li, 2010). However, the Area Quota Method needs to determine the specific distribution and water quota of different vegetation types. Determining the spatial distribution of different vegetation types is difficult to perform accurately over large areas. However, it is relatively easy to get the data required for the Diving Evaporation Method, such as the annual average depth of groundwater and evaporation, the latter determined using conventional meteorological evaporating dishes (https://data.cma.cn/). Therefore, the ecological water demand of vegetation in different ecological protection areas was calculated using the Diving Evaporation Method. The ecological water demand of vegetation (*Y*_ecology_) in dry areas was calculated as follows:

(1) $${Y_{\rm ecology}} = W = E\!\cdot\!A$$

where *E* is the evaporation intensity of diving water (Diving water is the groundwater buried in the first stable aquifer below the surface of the earth. Most of the groundwater that is usually seen is diving water), and *A* is the area of ecological protection obtained from the local watershed authorities, or by referring to the relevant literatures or reports. The parameter *E* was calculated as:

(2) $$E = {\rm a}{\left( {1 - H/{H_{\rm max}}} \right)^b}{E_{\rm \phi 20}}$$

In formula 2, *E* is the evaporation intensity of diving water; ${E_{\rm \phi 20}}$ is an observed value of evaporation from conventional meteorological dishes (obtained from the China Meteorological Science Data Sharing Service Website, https://data.cma.cn/); *H* is the annual average depth of groundwater, and *H*_max_ is the maximum depth of groundwater, with *a* and *b* as empirical coefficients, which were assigned values of 0.62, and 2.8, respectively. *H*_max_ and *H* was monitored by using XH17-S1 Telemetry Water Level Meter in the field. *a* and *b* can be obtained by consulting local watershed authorities. Using the diving evaporation method, ecological water requirements under the three protection targets have been calculated to be 11.32 × 10^8^ m^3^, 3.41 × 10^8^ m^3^, and 1.00 × 10^8^ m^3^, respectively.

##### The annual average TEEWRs

We calculated the annual average TEEWRs (Table 1) of each river reach based on the calculated BEEWR of each section, the annual average river loss ($\bar{X}_{\rm loss}$), and the ecological water requirement for vegetation in dry areas (*Y*_ecology_).

**Table 1:** The annual average TEEWRs for the five reaches under the three protection targets (unit: 10^8^m^3^) .

	Kaqun	Yiganqi	48th Regiment Ferry	Alektamu	Heiniyazi
The first protection target	26.39	23.61	17.67	15.35	11.32
The second protection target	18.48	15.70	9.76	7.44	3.41
The third protection target	25.24	21.62	13.88	10.85	4.30

It has been assumed that the difference between the annual average runoff $(\bar X)$ and *X*_irrigation and domestic use_ was used to meet ecological water requirements of natural vegetation, then, the calculation of *x*_loss_ (the corresponding river loss in the whole river for *Y*_ecology_):

(3) $${x_{\rm loss}} = {\bar X_{\rm loss}}\cdot {Y_{\rm ecology}}/(\bar X - {X_{\rm irrigation\; and\; domestic\; use}})$$

where *Y*_*ecology*_ is the ecological water demand of vegetation calculated by the Diving Evaporation Method; ${\bar X_{\rm loss}}$ is the annual average river loss along the entire river, as derived from Xinjiang Tarim River Basin Kashi Management Bureau; $\bar X$ is the annual average river runoff along the Kaqun reach (derived from the Xinjiang Tarim River Basin Kashi Management Bureau); and *X*_irrigation and domestic use_ is the amount of water diversion for irrigation and domestic use as determined by the Three Red Lines standard (the latter from the Xinjiang Tarim River Basin Kashi Management Bureau).

Based on the proportion between ${\bar X_{\rm loss}}$ in the whole river and river losses (${\bar x_{\rm loss}}$) along each river reach, the water loss along each river reach (*x*_ecology–loss_) was determined from *x*_loss_. Then, the different *Y*_ecology_ was reversed to obtain TEEWR for each section. Finally, the TEEWRs of the Kaqun reach were obtained as follows:

(4) $${\rm TEEWR} = {Y_{\rm ecology}} + \left( {{{\bar x}_{\rm loss}}\cdot {x_{\rm loss}}} \right)/{\bar X_{\rm loss}}$$

Target EEWR did not change with the amount of water in upstream reaches. Therefore, TEEWRs in each reach for the examined total annual flow frequencies were the same as the annual average TEEWRs.

#### Methods on calculating the runoff, the river loss, water requirement index in a given flow frequency

##### Determination of the runoff along the Kaqun reach

**Figure 2:**
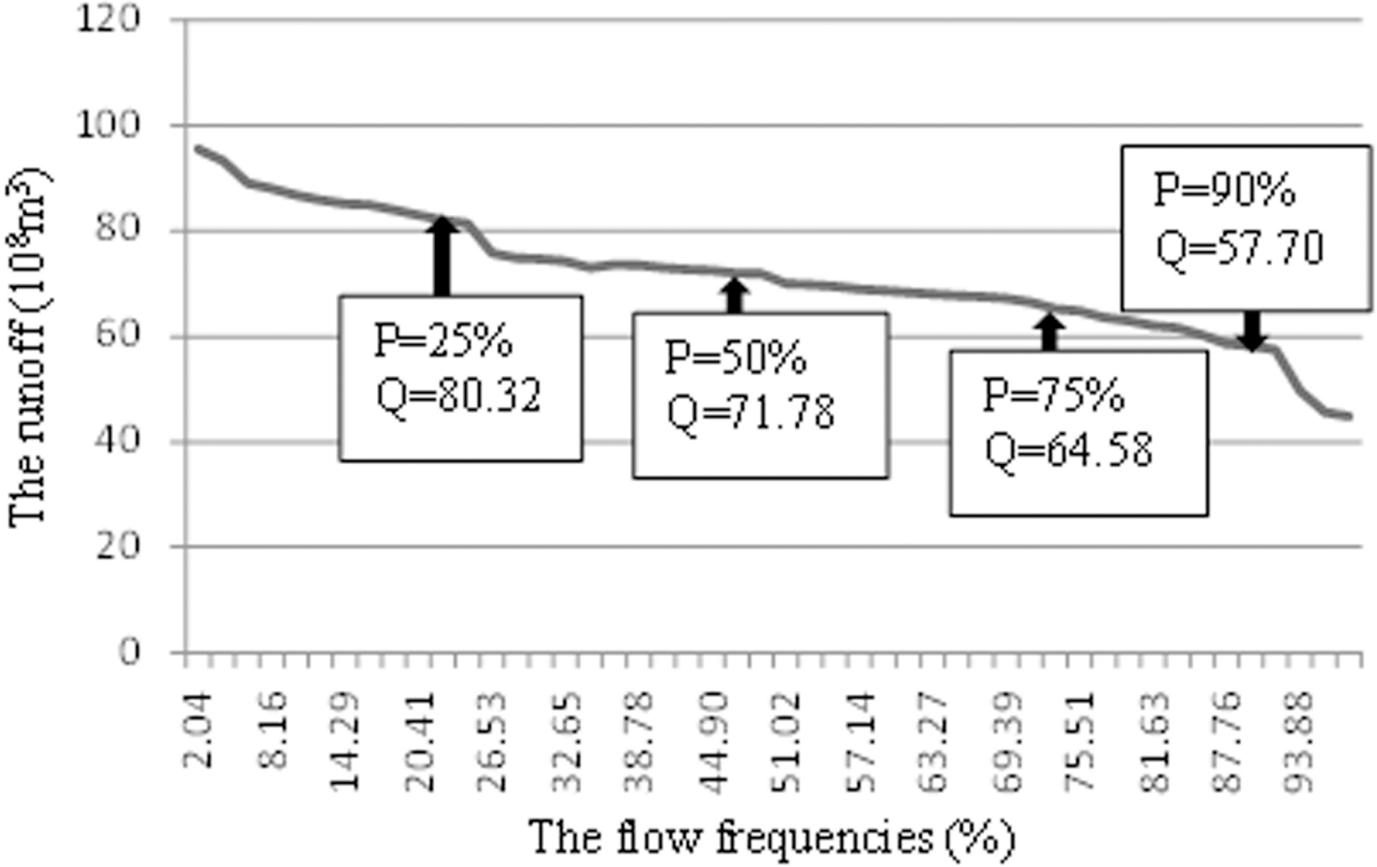
Frequency plot of annual runoff at the Kaqun Hydrographic Station of the Yarkand River.

A Log-Pearson Type III Frequency curve (P-III Frequency curve) was constructed for the Kaqun reach using runoff data from 1970 to 2017 (Fig. 2). Following the development of the curve, runoff data for different flow frequencies (25%, 50%, 75%, and 90%) were obtained, of which 25% are years of abundant water, 50% are flat years, and 75% and 90% are dry years and extremely dry years, respectively.

##### The river loss along each river reach

The formulas for determining *x_n_*_loss_ along each river reach for flows of a given return frequency were calculated as follows:

(5) ${{\hskip-12pt}\bar x_{n\; {\rm loss}}} = \displaystyle{{{{\bar X}_{\rm loss}}} \over {\bar X}}\cdot {x_n} (n: 25\%, 50\%, 75\%, 90\%)$

where ${\bar x_{n\; {\rm loss}}}$ is the river loss along the entire river for a given flow frequency; *n* is the flow frequency being considered; $\bar X$ is the annual average runoff at the Kaqun reach monitoring station (i.e., the runoff under the 50% flow frequency); ${\bar X_{\rm loss}}$ is the annual average river loss along the whole river (that is the river loss under the 50% flow frequency); and *x_n_* is the runoff along the Kaqun reach for a given flow frequency.

After ${\bar x_{n\; \rm loss}}$ was calculated, the river loss along each river reach for each of the defined flow frequencies (*x_n_*_loss_) was calculated using:

(6) $${x_{n\; {\rm loss}}} = \displaystyle{{{x_{50\% \; {\rm loss}}}} \over {{{\bar x}_{50\% \; {\rm loss}}}}}\cdot 100\% \cdot {\bar x_{n\; {\rm loss}}} = A\!\cdot\!{\bar x_{n\; {\rm loss}}}$$

where ${\bar x_{50\% \; {\rm loss}}}$ is the river loss along the whole river for the 50% flow frequency; *x_50%_*_loss_ is the river loss along each river reach for the 50% flow frequency; ${\bar x_{n\; {\rm loss}}}$ is the river loss along the whole river for a specific flow frequency; *x_n_*_loss_ is the river loss along each river reach for each flow water frequency; and $A$ is the ratio between ${x_{50\% \; {\rm loss}}}$ and ${\bar x_{50\% \; {\rm loss}}}$, that is, 18%, 39%, 15%, and 27% in Kaqun-Yiganqi, Yiganqi-48th Regiment Ferry, 48th Regiment Ferry-Alektamu, and Alektamu-Heiniyazi, respectively.

In this paper, $\bar X$ = 68.16 × 10^8^ m^3^ (derived from Xinjiang Tarim River Basin Kashi Management Bureau), ${\bar X_{\rm loss}}$ = 15.07 × 10^8^ m^3^ (derived from Xinjiang Tarim River Basin Kashi Management Bureau), *x_n_* = 80.32 × 10^8^ m^3^, 71.78 × 10^8^ m^3^, 64.58 × 10^8^ m^3^, and 57.70 × 10^8^ m^3^ for the 25%, 50%, 75% and 90% flow frequencies, respectively (obtained by analyzing the P-III frequency curve), and ${\bar x_{50\% \; {\rm loss}}}$ = ${\bar X_{\rm loss}}$ = 15.07 × 10^8^ m^3^ (derived from Xinjiang Tarim River Basin Kashi Management Bureau).

##### The amount of water (X_o_) along other river reaches

Combined with the runoff (*x_n_*) from the Kaqun reach for the different flow frequencies, the water diversion (*X*_irrigation and domestic use_) and withdrawal (*X_w_*) of water from each river reach, the loss of the interval river (*x*_loss_), and the amount of water (*X_o_*) from other study reaches can be obtained by the following formula:

(7) $${X_o} = {X_p} - {x_{n\; {\rm loss}}} - {X_{\rm irrigation\; and\; domestic\; use}} + {X_w}$$

where *X_o_* is the amount of water along one reach; *X*_*p*_ is the amount of water in a previous section; *x_n_*_loss_ is the interval river loss for the different flow frequencies; *X*_irrigation and domestic use_ is the “Three Red Lines” water diversion; and *X*_*w*_ is the water withdrawal. The “Three Red Lines” water plan for 2018 was used for *X*_irrigation and domestic use_ for the different flow frequencies.

##### Guaranteeing EEWRs for the different flow frequencies

The degree to which runoff along the different reaches met the BEEWR and TEEWRs was determined by calculating Water Requirement Index (WRI). The specific calculation method is: WRI = (*X_o_*/BEEWR or TEEWR) × 100%. If WRI >100%, the amount of water at the monitoring station cross section is high for BEEWR or TEEWRs. If WRI < 100%, the amount of water at the cross section is low for BEEWR or TEEWRs.

For the determination of WRI of BEEWR, the runoff for the different flow frequencies was directly compared with BEEWR, and the calculation of WRI was obtained. After comparison with the first, second and third TEEWRs, WRI was calculated. Overall, for a given flow frequency, when WRI was less than 100% along a given reach, that frequency of flow could not meet the TEEWR.

## Results and Analysis

### Variation in runoff along the Kaqun reach

**Figure 3:**
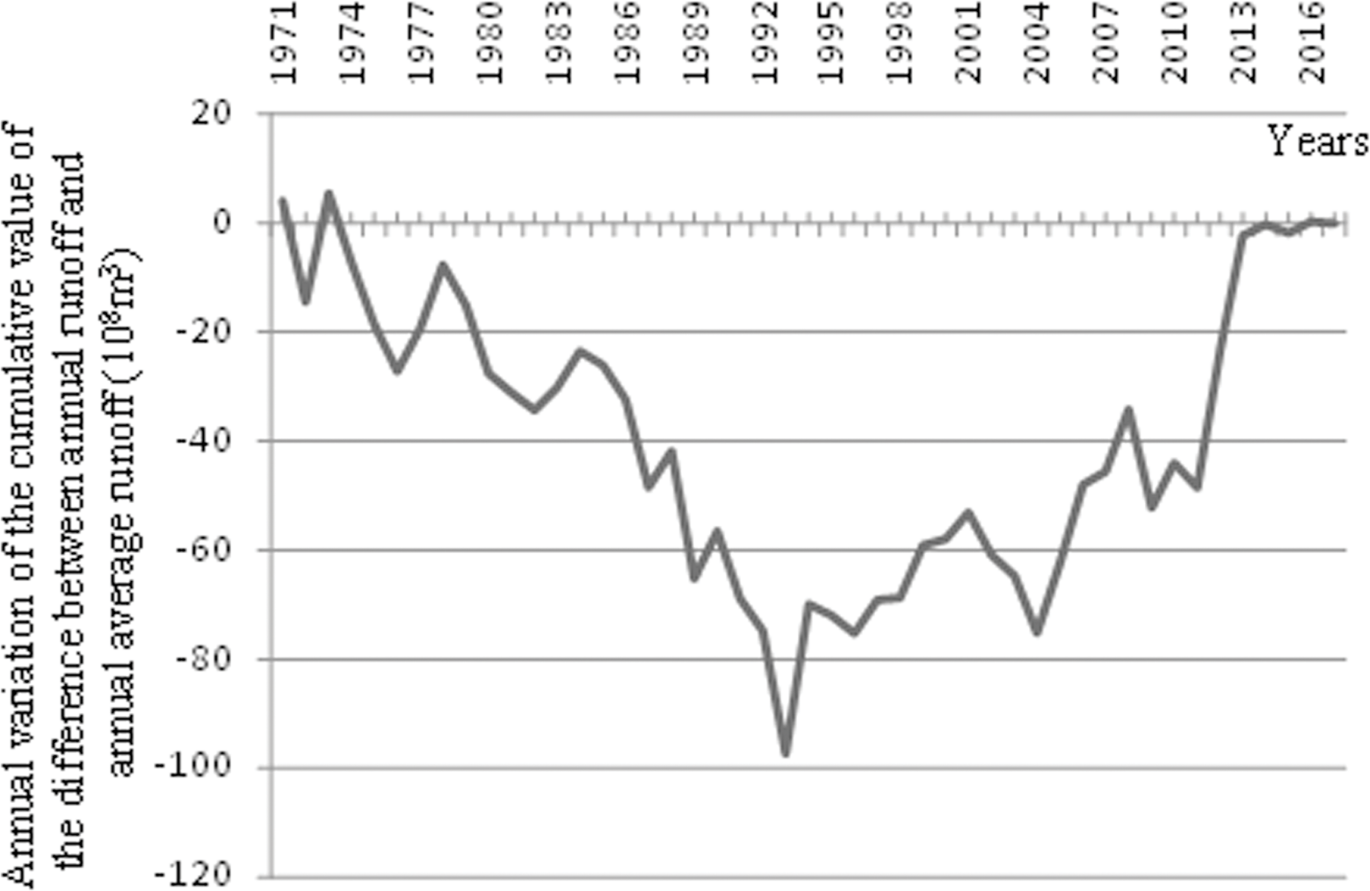
Annual variation of the cumulative value of the difference between annual runoff and annual average runoff at the Kaqun Hydrographic Station of the Yarkand River.

Figure 3 provides the change in vertical cumulative curve on the yearly cumulative values of the difference between the runoff in each year and the multi-year average runoff within the Kaqun during the past 48 years (1970–2017). Figure 3 shows that flow at the Kaqun Hydrographic Station from 1970 to 2017 can be divided into three phases: (1) a dry phase from 1970 to 1992; (2) a wet phase from 1993 to 2004; and (3) a moderately wet phase from 2005 to 2017. Table 2 shows the statistics and *T*-test results of the runoff differences among the three defined periods. From Table 2, it can be seen that there were significantly difference among these three periods with the significance value less than 0.05.

**Table 2:** Statistics and *T*-test in runoff for the three time intervals at the Kaqun Station of the Yarkand River (unit: 10^8^ m^3^).

Time stages	Average annual runoff (10^8^ m^3^)	Standard deviation	Standard error mean	Sig. (two-tailed)	95% Confidence interval of the difference	
					Lower	Upper
1970–1992	64.91	10.95	2.28	0.00	60.17	69.64
1993–2004	68.12	12.10	3.49	0.00	60.43	75.81
2005–2017	73.93	11.43	3.17	0.00	67.02	80.84

Within the each of the three time intervals, there are small fluctuations in runoff, that is, there is a transition from wet to dry conditions (Fig. 3). Using the time-series of annual runoff at the Kaqun Hydrographic Station, the annual frequency of runoff over past 48 years was analyzed. The frequency of runoff for the three time intervals was then determined for *P* = 25%, 50%, 75%, and 90%, and the runoff were 80.32 × 10^8^ m^3^, 71.78 × 10^8^ m^3^, 64.58 × 10^8^ m^3^, and 57.70 × 10^8^ m^3^, respectively (Fig. 2). During slightly dry years (0.63 < *P* < 0.87), the average annual runoff was less than 70 × 10^8^ m^3^; however, when it was especially dry (*P* > 0.87), the average annual runoff was less than 60 × 10^8^ m^3^.

### Guarantee status of BEEWR and TEEWRs for different hydrological frequencies

#### Guarantee of BEEWR along the Kaqun reach

The BEEWR for the non-flood season (October–May of the following year), the flood season (June–September) and the entire year were determined by the Tennant method (Mishra, Amrit & Pandey, 2019); BEEWRs were 10.90 × 10^8^ m^3^, 1.18 × 10^8^ m^3^ and 12.08 × 10^8^ m^3^, respectively. Using the multi-year runoff record from the Kaqun Hydrographic Station, the calculated runoff corresponding to 25%, 50%, 75%, and 90% annual runoff frequencies were 80.32 × 10^8^ m^3^, 71.78 × 10^8^ m^3^, 64.58 × 10^8^ m^3^, and 57.70 × 10^8^ m^3^, respectively. It is worth noting that they are all larger than the annual BEEWRs, that is, both the annual flow during dry and wet years meet the BEEWR standard at the Kaqun monitoring station.

#### Guarantee of BEEWR at the other sections

*X*_*o*_ for different flow frequencies along the other sections was determined by considering *X*_irrigation and domestic use_ and *x*_loss_, plus *X_w,_* in comparison to BEEWR for each reach. This calculation did not take TEEWRs into account (Table 3). The four reaches met the standard for an annual BEEWR at the four considered flow frequencies.

**Table 3:** The amount of water and the evaluation of WRI for the BEEWRs (unit: 10^8^ m^3^).

Flow frequencies	25% (Wet year)	50% (Moderate wet year)	75% (Dry year)	90% (Extremely dry year)	BEEWRs
Runoff in Kaqun	80.32	71.78	64.58	57.70	12.08 (the whole year)
WRI	664.90	594.21	534.60	477.65	
Water diversion (*X*_irrigation and domestic use_)	34.59	34.59	34.59	34.59	
Water withdrawal (*X*_*w*_)	9.27	9.27	9.27	9.27	
River loss (*x*_loss_)	3.22	2.78	2.48	2.23	
Runoff in Yiganqi	51.78	43.68	36.78	30.15	9.30 (the whole year)
WRI	556.70	469.68	395.48	324.19	
Water diversion(*X*_irrigation and domestic use_)	14.91	14.91	14.91	14.91	
Water withdrawal (*X_w_*)	3.66	3.66	3.66	3.66	
River loss (*x*_loss_)	6.87	5.94	5.31	4.77	
Runoff in 48th Regiment Ferry	33.66	26.49	20.22	14.13	2.71 (April–September)
WRI	1242.00	977.49	746.13	521.40	
Water diversion (*X*_irrigation and domestic use_)	1.54	1.54	1.54	1.54	
Water withdrawal (*X_w_*)	0.00	0.00	0.00	0.00	
River loss (*x*_loss_)	2.68	2.32	2.07	1.86	
Runoff in Alektamu	29.44	22.63	16.61	10.73	1.19 (June, September)
WRI	2473.00	1901.68	1395.80	901.68	
Water diversion (*X*_irrigation and domestic use_)	0.00	0.00	0.00	0.00	
Water withdrawal (*X_w_*)	0.00	0.00	0.00	0.00	
River loss (*x*_loss_)	4.66	4.03	3.60	3.24	
Runoff in Heiniyazi	24.78	18.60	13.01	7.49	0.44 (September)
WRI	5631.00	4227.27	2956.82	1702.27	

#### Guarantee of TEEWRs

A total flow of 51.43 × 10^8^ m^3^ was associated with *X*_irrigation and domestic use_ for all the other reaches. It was distributed proportionately such that *X*_irrigation and domestic use_ along the Kaqun-Yiganqi, Yiganqi-48th Regiment Ferry, and 48th Regiment Ferry-the Alektamu reaches were 34.82 × 10^8^ m^3^, 15.02 × 10^8^ m^3^ and 1.54 × 10^8^ m^3^, respectively. Using the ratio between the annual average actual water diversion (*X*_irrigation and domestic use_) in each river reach and the annual average total water diversion (*x*, derived from Xinjiang Tarim River Basin Kashi Management Bureau) in the entire river, and assuming that BEEWR, *X_w_* and *X*_loss_ corresponding to the different flow frequencies were unchanged, after runoff in Kaqun under the different flow frequencies is deducted from *X*_irrigation and domestic use_, BEEWR and *X*_loss_ in the irrigation area, respectively, plus *X_w_*, the remaining water volume from the different river reaches was compared with TEEWRs (Table 4), the results can be seen: (1) at 25% and 50% flow frequencies, the runoff along each reach can meet BEEWR as well as the second and third TEEWRs; (2) for an annual runoff level of 75% of the flow frequency, the BEEWR is met, along with the third TEEWR for each reach throughout the year; however, the available flow cannot meet the water requirements for ecologically sensitive areas; (3) for a flow frequency of 90%, the available runoff can only meet the BEEWR of each reach; it cannot meet the requirements of the first, second and third TEEWRs. Runoff of less than 75% of the incoming flow frequency can meet the BEEWR of the river; the remaining water can meet the water demand of 1.00 × 10^8^ m^3^ in the key protected area of *P. euphratica* and 3.30 × 10^8^ m^3^ for the main stream of the Tarim River.

**Table 4:** The amount of water, BEEWR, TEEWRs, and the evaluation of WRI on the TEEWRs, for different flow frequencies in the Yarkand River (unit: 10^8^ m^3^). WRI 1 indicates whether the amount of water flowed from the previous section to the next section after water diversion, water withdrawal, and river loss to meet the BEEWRs; WRI 2 indicates whether the amount of water flowed from the previous section to the next section after water diversion, water withdrawal, and river loss to meet the BEEWR and the third TEEWR; WRI 3 indicates whether the amount of water flowed from the previous section to the next section after water diversion, water withdrawal, and river loss to meet the BEEWR, the second and third TEEWRs; WRI 4 indicates whether the amount of water flowed from the previous section to the next section after water diversion, water withdrawal, and river loss to meet the BEEWR, the first, second and third TEEWRs.

Flow frequencies	25% (Wet year)	50% (Moderate wet year)	75% (Dry year)	90% (Extremely dry year)	BEEWR	The third TEEWR	The second TEEWR	The first TEEWR	The total TEEWR	Remarks
Runoff in Kaqun	80.32	71.78	64.58	57.70	12.08	19.37	18.48	26.39	76.32	The whole year
WRI 1	664.90	594.21	534.61	477.65						Considering BEEWRs
WRI 2	255.39	228.24	205.34	183.47						Considering BEEWR and the third TEEWR
WRI 3	160.87	143.76	129.34	115.56						Considering BEEWR, the second and third TEEWRs
WRI 4	105.24	94.05	84.62	75.60						Considering BEEWR, the first, second and third TEEWRs
Water diversion (*X*_irrigation and domestic use_)	34.82	34.82	34.82	34.82						
Water withdrawal (*X*_*w*_)	9.27	9.27	9.27	9.27						
River loss (*x*_loss_)	3.22	2.78	2.48	2.23						
Runoff in Yiganqi	51.55	43.45	36.55	29.92	9.3	16.59	15.7	23.61	65.2	The whole year
WRI 1	554.30	467.20	393.01	321.72						Considering BEEWRs
WRI 2	199.11	167.83	141.17	115.57						Considering BEEWR and the third TEEWR
WRI 3	123.95	104.47	87.88	71.94						Considering BEEWR, the second and third TEEWRs
WRI 4	79.06	66.64	56.06	45.89						Considering BEEWR, the first, second and third TEEWRs
Water diversion (*X*_irrigation and domestic use_)	15.02	15.02	15.02	15.02						
Water withdrawal (*X*_*w*_)	3.66	3.66	3.66	3.66						
River loss (*x*_loss_)	6.87	5.94	5.31	4.77						
Runoff in 48th Regiment Ferry	33.32	26.15	19.88	13.79	2.71	10.65	9.76	17.67	40.79	April–September
WRI 1	1229.5	964.94	733.58	508.86						Considering BEEWRs
WRI 2	249.40	195.73	148.80	103.22						Considering BEEWR and the third TEEWR
WRI 3	144.12	113.11	85.99	59.65						Considering BEEWR, the second and third TEEWRs
WRI 4	81.69	64.11	48.74	33.81						Considering BEEWR, the first, second and third TEEWRs
Water diversion (*X*_irrigation and domestic use_)	1.54	1.54	1.54	1.54						
Water withdrawal (*X_w_*)	0	0	0	0						
River loss (*x*_loss_)	2.68	2.32	2.07	1.86						
Runoff in Alektamu	29.10	22.29	16.27	10.39	1.19	8.33	7.44	15.35	32.31	June and September
WRI 1	2445.3	1873.11	1367.2	873.11						Considering BEEWRs
WRI 2	241.69	185.13	135.13	86.30						Considering BEEWR and the third TEEWR
WRI 3	171.58	131.43	95.93	61.26						Considering BEEWR, the second and third TEEWRs
WRI 4	90.06	68.99	50.36	32.16						Considering BEEWR, the first, second and third TEEWRs
Water diversion (*X*_irrigation and domestic use_)	0	0	0	0						
Water withdrawal (*X_w_*)	0	0	0	0						
River loss (*x*_loss_)	4.66	4.03	3.6	3.24						
Runoff in Heiniyazi	24.44	18.26	12.67	7.15	0.44	3.3	3.41	11.32	18.47	September
WRI 1	5554.50	4150.00	2879.50	1625.00						Considering BEEWRs
WRI 2	653.48	488.24	338.77	191.18						Considering BEEWR and the third TEEWR
WRI 3	341.82	255.39	177.20	100.00						Considering BEEWR, the second and third TEEWRs
WRI 4	132.32	98.86	68.60	38.71						Considering BEEWR, the first, second and third TEEWRs

## Discussion

### Major ecological protection targets

Major ecological protection targets for the different flow frequencies have been determined. The remaining water is important to meet the different TEEWRs after first meeting *X*_irrigation and domestic use_, BEEWR and *x*_loss_ for the runoff. However, the maximum irrigation and domestic use (i.e., *X*_irrigation and domestic use_) in the oasis of the arid area should not exceed the “Three Red Lines” standard. Furthermore, BEEWR and *x*_loss_ need to be met because maintaining the ecological function of the river itself and the health of the aquatic ecosystem is essential. The remaining water should be used to meet other ecological needs as much as possible. The average annual runoff of rivers between 2000 and 2016 was 72.04 × 10^8^ m^3^, *X*_irrigation and domestic use_ of irrigation areas was 51.43 × 10^8^ m^3^, *X*_*w*_ was 12.39 × 10^8^ m^3^, and ${\bar X_{\rm loss}}$ was 15.07 × 10^8^ m^3^. BEEWR for the entire year within the Kaqun reach was 12.08 × 10^8^ m^3^. When the average annual runoff of the Yarkand River is subtracted from *X*_irrigation and domestic use_, *x_loss_* and BEEWRs, plus *X_w_*, the remaining water volume was 6.85 × 10^8^ m^3^. During the past 17 years, the average flow of water from the Yarkand River has been above that associated with a moderately wet year, and the runoff was relatively high. After satisfying the *X*_irrigation and domestic use_ in the irrigation area, *x*_loss_, and the BEEWRs, the remaining water met the third TEEWR. Thus, it is suggested that in the Yarkand River Basin, only the protection of the key *P. euphratica* area and 3.30 × 10^8^ m^3^ of ecological water to the mainstream of the Tarim River should be the main ecological protection goals of the basin.

### Water resource allocation strategy

Regarding the allocation of water resources in the Yarkand River Basin, Yu (2002) studied the optimal distribution ratio of water resources between irrigation, ecological water use, and water use for power generation. They argued that the water resources of the Yarkand River Basin first need to meet the needs of industrial and agricultural irrigation and domestic use, along with domestic needs of 46.40 × 10^8^ m^3^ in the basin. The available water should also be used to meet the needs of downstream ecological water requirements and the water supply of the Tarim River (12.06 × 10^8^ m^3^). The remaining water was for water storage of the plain reservoir in the upstream and middle reaches. This study did not consider BEEWR and *x_loss_* of the river. If BEEWR and *x_loss_* of the river are considered, the amount of water allocated to agricultural and industrial production, domestic needs, and ecology by previous studies is unreasonable. Li (2011) also studied the allocation of water resources in the Yarkand River Basin. He concluded that it was necessary to first meet the task of transporting 3.30 × 10^8^ m^3^ of ecological water to the Tarim River from July to September, and secondly to meet the water use and power generation needs in the irrigation area. Compared with the results of Yu (2002), the ecological water allocation to the main stream of the Tarim River was relatively reasonable, but BEEWR and *x_loss_* of the river were not considered, too. No research was conducted on the runoff and water demand of the river at different flow frequencies. This paper not only calculated BEEWRs and *x_loss_* along the Yarkand River course, but also systematically considered the ecological requirements of natural vegetation in different areas. The changes of river runoff for different flow frequencies and the degree to which water demands for natural vegetation were met were also considered. Based on total *X*_irrigation and domestic use_ (calculated according to the “Three Red Lines” standards), actual annual average *X_w_*, *x*_loss_, and BEEWR and TEEWRs for different flow frequencies, the guarantee degree for different river runoff for BEEWR and TEEWRs was evaluated, and a water resource allocation strategy was proposed. In this paper, the water resource allocation strategy for the basin for different flow frequencies (in order of priority) was as follows: (1) at any water frequency, meet *X*_irrigation and domestic use_ of the irrigation area not exceeding the Three Red Lines standard; (2) meet the BEEWR of 12.08 × 10^8^ m^3^ along the Kaqun reach; (3) meet *x*_loss_ of the Kaqun-Heiniyazi reach under different flow frequencies; and (4) use the remaining water to meet the second and third TEEWRs during wet years, and the third TEEWR during moderately wet years. During dry and extremely dry years, only the BEEWR of the river can be met, and only in the flood season, not to meet the third TEEWR.

## Conclusions

In this article, the Yarkand River, a typical inland river basin in China characterized by extreme drought, was selected to study the EEWRs for different flow frequencies. The following conclusions were reached by the current study:

1. The total annual flow frequencies, including *P* = 25%, 50%, 75%, and 90%, were found to be 80.32 × 10^8^ m^3^, 71.78 × 10^8^ m^3^, 64.58 × 10^8^ m^3^, and 57.70 × 10^8^ m^3^, respectively. When the year was slightly dry (0.63 < *P* < 0.87), the average annual runoff was less than 70×10^8^ m^3^. By contrast, when it was an especially dry year (*P* > 0.87), the average annual runoff was less than 60 × 10^8^ m^3^.
2. After *X*_irrigation and domestic use_ in the irrigation area, *x*_loss_, BEEWR was subtracted from the runoff volume at the Kaqun reach, and plus *X*_*w*_, and the remaining water volume during a wet year was able to meet the second and third TEEWRs. The remaining water during a moderately wet year could meet the third TEEWR; however, during dry and extremely dry years, there was little or no residual water available to meet TEEWRs.
3. The water resource allocation strategy of the basin (in order of priority) is as follows: (A) at any flow frequency, *X*_irrigation and domestic use_ in the irrigation area should not exceed the “Three Red Lines” first; (B) a BEEWR of 12.08 × 10^8^ m^3^ in the Kaqun section should then be met; and (C) *x*_loss_ of the Kaqun-Heiniyazi section for the different flow frequencies should be met next. Finally, (D) depending on the amount of remaining water, the second and third TEEWRs can be fulfilled during wet years, and the third TEEWR can be met during moderately wet years. During dry and extremely dry years, the BEEWR of the river can be met only during the flood season; there is no need to meet the third TEEWR.

## Supplemental Information

10.7717/peerj.8285/supp-1 Supplemental Information 1 Flow in Kaqun section, the annual average basic and target EEWR, water diversion and withdrawal.

## Additional Information and Declarations

### Data Availability

The following information was supplied regarding data availability: The raw measurements are available in the Supplemental Files.
